# Synergies reciprocally relate end-effector and joint-angles in rhythmic pointing movements

**DOI:** 10.1038/s41598-019-53913-9

**Published:** 2019-11-22

**Authors:** Tim A. Valk, Leonora J. Mouton, Egbert Otten, Raoul M. Bongers

**Affiliations:** Center for Human Movement Sciences, University of Groningen, University Medical Center Groningen, Antonius Deusinglaan 1, 9713 AV Groningen, The Netherlands

**Keywords:** Motor control, Human behaviour

## Abstract

During rhythmic pointing movements, degrees of freedom (DOF) in the human action system—such as joint-angles in the arm—are assumed to covary to stabilise end-effector movement, e.g. index finger. In this paper, it is suggested that the end-effector movement and the coordination of DOF are reciprocally related in synergies that link DOF so as to produce the end-effector movement. The coordination of DOF in synergies and the relation between end-effector movement and DOF coordination received little attention, though essential to understand the principles of synergy formation. Therefore, the current study assessed how the end-effector movement related to the coordination of joint-angles during rhythmic pointing across target widths and distances. Results demonstrated that joint-angles were linked in different synergies when end-effector movements differed across conditions. Furthermore, in every condition, three joint-angles (shoulder plane of elevation, shoulder inward-outward rotation, elbow flexion-extension) largely drove the end-effector, and all joint-angles contributed to covariation that stabilised the end-effector. Together, results demonstrated synergies that produced the end-effector movement, constrained joint-angles so that they covaried to stabilise the end-effector, and differed when end-effector movement differed. Hence, end-effector and joint-angles were reciprocally related in synergies—indicating that the action system was organised as a complex dynamical system.

## Introduction

A key question in the domain of motor control regards the principles on which the coordination of the abundant number of degrees of freedom (DOF) in the human action system—e.g. joints-angles or muscles in the arm—during the performance of goal-directed end-effector movements—e.g. index finger movements—is based. According to the dynamic systems approach to movement coordination, DOF are coordinated in synergies, reflected in the temporary linking of DOF causing these linked DOF to act as a functional, coherent unit^1–5^. This notion of synergies is around for several decades and is used in experimental as well as in theoretical studies (e.g., Turvey^3^ and Kelso^4^ provided for overviews). Experimental studies examining synergies from this approach have mainly focused on the characteristics of synergies as revealed in rhythmic end-effector movements^e.g.^^6–8^. The examination of the coordination of DOF in synergies that produces these end-effector movements has received little attention. Yet, due to the intrinsic connections among all levels of the action system, the coordination of DOF is suggested to be reciprocally related to the production of end-effector movements^3,5,9,10^, in which the synergy provides for this reciprocal relation. A thorough understanding of the principles of synergy formation with which end-effector movements are produced thus also requires the examination of the coordination of DOF as well as its relation with end-effector movement. Therefore, the current study examined how end-effector movement related to the coordination of joint-angles—selected as DOF—in rhythmic pointing to assess the suggested reciprocal relation between these two levels.

From the point of view of the dynamic systems approach, a goal-directed end-effector movement emerges from self-organisation processes driven by the interaction among constraints in individual, task, and environment that act on the action system^1,11–13^. For instance, target width and target distance are suggested to act as task constraints during pointing movements^14–17^. Based on these interactions, a synergy emerges from the DOF, in which the synergy is characterised by an attractor^10,18,19^. Changes in one or more constraints can lead to a shift in parameter settings of the attractor or a transition to a new attractor, which both can be reflected in the end-effector movement. For instance, in rhythmic pointing movements, it has been shown that characteristics of a limit-cycle oscillator—i.e., an attractor—describing the kinematic pattern of the end-effector movement systematically differed with different target widths and target distances^14–17^. The resultant kinematic pattern of the end-effector leads to a specific movement time^20,21^ and velocity profile of the end-effector^22^ for a given target width and target distance combination (Plamondon & Alimi^23^ provide for an overview of this Fitts’ paradigm). The question examined in the current study is how, across these task constraints, joint-angles are coordinated in synergies and how this relates to the differences in end-effector movement in rhythmic pointing. Note that the generality of findings with just rhythmic movements can be questioned from the notion that discrete and rhythmic movements are controlled differently^24–26^. In the current study, we employed a method that is usually employed in discrete upper extremity movements^27–38^ to assess the coordination of DOF in synergies in rhythmic movements, allowing to indicate whether differences between discrete and rhythmic movements also show up at the synergy level.

In the experiment, participants made rhythmic pointing movements between two targets, in which we manipulated index of difficulty (ID; a logarithmic function of target width and target distance^20,21^) and target distance across conditions to elicit differences in end-effector movements as reported previously^14–17^. As a synergy is assumed to reciprocally relate end-effector and DOF—e.g. joint-angles—this implies that the end-effector movement that emerges from the synergy linking joint-angles in turn constrains the way in which joint-angles are coordinated in this synergy^1,3,5,9,10^. Therefore, if end-effector and joint-angles are reciprocally related in synergies, differences at the level of the end-effector^cf.^^14–17^ can be expected to be associated with differences in joint-angle coordination, and vice versa. Both end-effector movements and joint-angle coordination can obviously be expected to be different when task constraints differ more than as employed in the current study—e.g. for pointing in different directions. However, in that case the question will be whether these differences result from i) the reciprocal relation between end-effector and joint-angles, or ii) the large variation in task constraints. In the current task, within every target distance, the ID of the targets was manipulated (by adjusting the width of the targets with respect to the distance between targets) while the location of the centre of the targets stayed the same. This implied that the expected differences in end-effector movement across IDs could in principle be produced with the same joint-angle coordination patterns, i.e. the same synergies. Therefore, findings of differences in joint-angle coordination across target widths will indicate that end-effector and joint-angles are reciprocally related in synergies.

Previous studies have shown that the linking among DOF^39,40^ or movements of individual DOF^41,42^ differed when end-effector movements across task constraints differed. For instance, during rhythmic pointing between two targets—the same paradigm as used in the current study—Vernooij *et al*.^39^ found that both the coordination patterns among muscles as well as the kinematic pattern of the end-effector differed across IDs. Their result showed that the differences in end-effector movement across task constraints were accompanied by differences in the synergistic behaviour of muscles. Although their study showed differences at both end-effector level and DOF level across conditions, the reciprocal relation between these levels of analysis was not addressed. Therefore, in the current study, we extended previous findings by directly assessing the reciprocal relation between the end-effector movement and joint-angles. To assess this reciprocal relation, we examined whether across conditions (i) synergies that stabilised the end-effector movement differed, and (ii) in these synergies, individual joint-angles had different roles in producing the end-effector movement.

As prerequisites to answer the questions of the current study, we first confirmed that the end-effector movement differed across conditions as reported previously^14–17^ and that joint-angles were linked in synergies during rhythmic pointing. Subsequently, based on the location in joint-space at which joint-angle configurations emerged across conditions, we examined whether synergies that stabilised the end-effector movement differed across conditions. Last, roles of individual joint-angles were assessed across conditions by examining (i) the coupling between the end-effector and individual joint-angles, (ii) the coupling between joint-angles, and (iii) the contribution of joint-angles to the covariation stabilising end-effector movements.

## Results

### Does the end-effector movement differ across task constraints?

**Figure 1:**
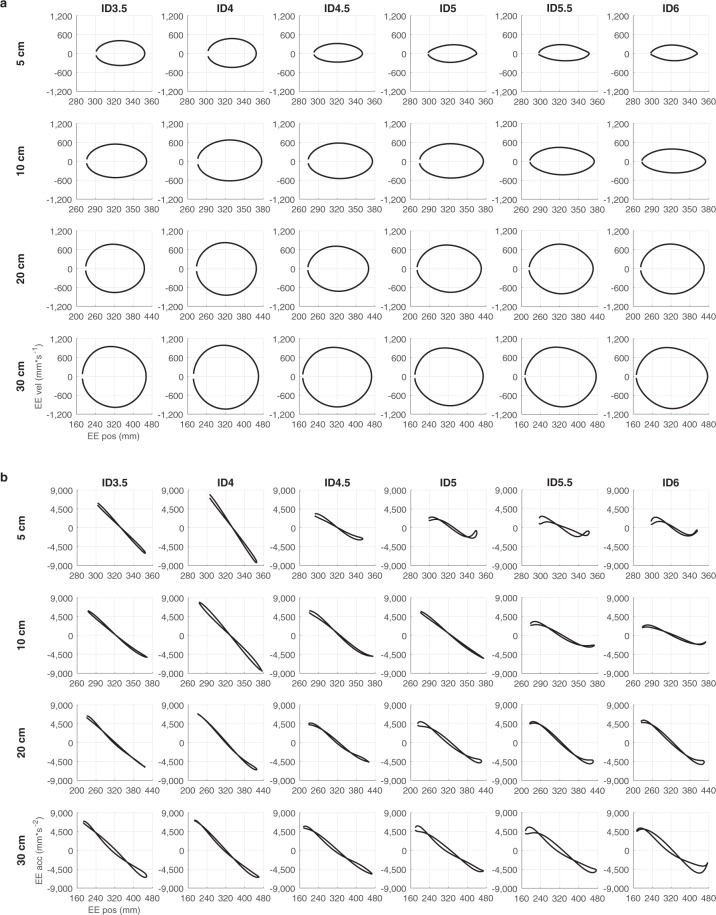
Example of phase portraits (end-effector position vs. end-effector velocity) **(a)** and Hooke’s portraits (end-effector position vs. end-effector acceleration) **(b**) across IDs and target distances, for one representative participant.

To examine whether end-effector movement differed across task constraints as presented in the various conditions, we first visually perused phase portraits (end-effector position vs. end-effector velocity) and Hooke’s portraits (end-effector position vs. end-effector acceleration) of all participants (an example is presented in Fig. 1). These portraits demonstrated different trajectories for different conditions. For instance, Hooke’s portraits showed a systematic deviation from a straight line to a characteristic N-shape when comparing conditions with lower IDs with that of higher IDs (Fig. 1), indicating that the limit-cycle attractor describing end-effector movement had more nonlinear stiffness for conditions involving higher IDs.

These visually observed patterns were supported by the examination of two objective measures. First, we assessed whether the percentage of variance that can be attributed to nonlinear components (NL) of the end-effector movement differed across conditions for the first and second cycle half of the pointing movements, by examining the deviation of the trajectory in the Hooke’s portrait from a straight line^cf.^^14^. Because NL-values were non-normally distributed, logarithmically transformed NL-values (NL_log_) were analysed. Results showed that for pointing movements involving higher IDs higher values of NL_log_ were observed, indicating more nonlinearity for higher IDs (F_2.72;46.25_ = 37.26, *p* < 0.001, *η*²_G_ = 0.30; mean [SD] NL_log_: ID3.5 = −5.6 [1.4], ID4 = −5.4 [1.3], ID4.5 = −4.9 [1.5], ID5 = −4.4 [1.3], ID5.5 = −3.9 [1.3], ID6 = −3.3 [1.2]; mean [SD] NL-values: ID3.5 = 0.8 [1.0]%, ID4 = 1.0 [1.4]%, ID4.5 = 1.5 [1.9]%, ID5 = 2.5 [3.2]%, ID5.5 = 4.1 [5.2]%, ID6 = 6.8 [7.7]%). Post-hoc pair-wise comparisons showed that ID3.5-4 differed from ID5-6 (all *p* < 0.001), ID4.5 differed from ID5.5-6 (all *p* < 0.001), and ID5-5.5 differed from ID6 (all *p* < 0.05). Also, for shorter target distances, the differences in NL_log_ between IDs was higher (F_5.76;97.98_ = 4.07, *p* < 0.005; *η*²_G_ = 0.07).

Second, we assessed the difference in the ratio between acceleration and deceleration time (AD_ratio_; a value of 1 means perfect symmetry) across conditions for the first and second cycle half of the pointing movements. For this measure, lower AD_ratio_-values were observed for pointing movements involving higher IDs (F_2.44;41.42_ = 13.16, *p* < 0.001, *η*²_G_ = 0.07; mean [SD] AD_ratio_: ID3.5 = 1.00 [0.12], ID4 = 0.97 [0.11], ID4.5 = 0.98 [0.14], ID5 = 0.96 [0.15], ID5.5 = 0.93 [0.15], ID6 = 0.90 [0.16]). Post-hoc pair-wise comparisons showed that ID3.5-4.5 differed from ID5.5-6 (all *p* < 0.05), and that ID5 differed from ID6 (*p* < 0.05). Furthermore, for the longer target distances, the downward trend in AD_ratio_ across IDs was more gradual, whereas for the shorter target distances a more fluctuating downward trend was observed (F_6.27;106.52_ = 2.48, *p* < 0.05; *η*²_G_ = 0.03). This interaction effect was present stronger in the second cycle half of the pointing movements than in the first cycle half (F_7.85;133.36_ = 4.41, *p* < 0.001; *η*²_G_ = 0.03). Last, in the first cycle half pointing movements were less symmetric than second cycle half (F_1;17_ = 30.74, *p* < 0.001, *η*²_G_ = 0.24; mean [SD] AD ratio: first cycle half = 0.89 [0.12], second cycle half = 1.02 [0.13]). No other effects were significant. Together, these results showed that the end-effector movement became more nonlinear when pointing movements involved higher IDs, with some differences in trends across IDs for the different target distances.

### Are joint-angles linked in different synergies so as to stabilise differences in end-effector movement across task constraints?

#### Synergistic linking among joint-angles

Before assessing whether joint-angles were linked in different synergies during the production of different end-effector movements across task constraints, we examined whether joint-angles were linked in synergies during the pointing movements. To assess such synergistic linking among joint-angles, we used the uncontrolled manifold (UCM) method^43–45^ which reveals the extent of covariation between joint-angles—considered as a hallmark for synergistic linking of DOF^cf.^^13^. The UCM method partitions the total variance in joint-angles across cycles in a part of covariance among joint-angles that stabilises the end-effector on its mean position (V_UCM_) and a part of error variance in joint-angles that leads end-effector positions to deviate from its mean (V_ORT_). As long as V_UCM_ exceeds V_ORT_, it is assumed that joint-angles are linked in synergies in which joint-angles covary so as to stabilise the end-effector movement^43–45^. We applied this method in every condition, across four phases of both the first and second cycle half (i.e. 0–25%, 26–50%, 51–75%, 76–100%).

**Figure 2:**
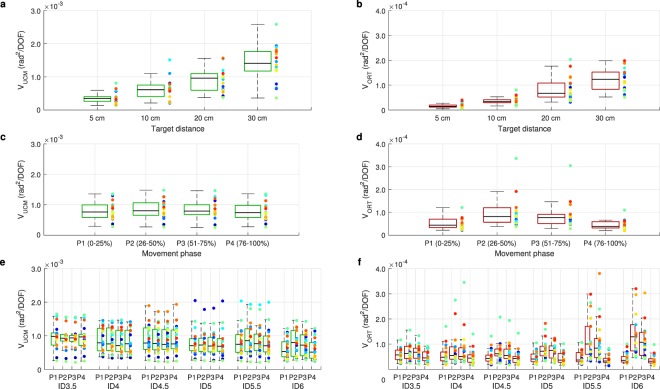
UCM variables V_UCM_ **(a**,**c**,**e)** and V_ORT_ **(b**,**d**,**e)**, as averaged across cycle halves, across target distance **(a**,**b)** and movement phase **(c**,**d)**, and IDs for separate movement phases **(e**,**f)**. Labels panels (**e**,**f**) P1: 0–25%, P2: 26–50%, P3:51–75%, P4: 76–100%. In all panels, coloured dots represent data of individual participants.

Results showed that in all conditions, V_UCMlog_ was larger than V_ORTlog_ (Fig. 2, Table 1; logarithmically transformed values of V_UCM_ and V_ORT_ were used to correct for nonnormality of the data). Furthermore, pointing movements involving shorter target distances exhibited higher amounts of joint-angle variance (Fig. 2, Table 1), in which post-hoc pair-wise comparisons showed that all target distances differed from each other (all *p* < 0.001). The interaction effect between variance type and target distance showed that the increase across distances was somewhat larger for V_ORTlog_ than V_UCMlog_. Moreover, the joint-angle variance was higher during the middle phases of the movement than during the first and last phase of the movement (Table 1, post-hoc pair-wise comparisons all *p* < 0.05). This lower joint-angle variance at the first and last phase primarily originated from V_ORTlog_, as indicated by the interaction between variance type and movement phase. Last, pointing movements involving higher IDs showed less variance during the first and last phases of the movement, but more variance during the middle phases of the movement, as compared to pointing movements involving low IDs.

**Table 1:** Main and interaction effects for the repeated-measures ANOVAs on V_UCMlog_ and V_ORTlog_.

Statistical analysis	Within-subject factor		Mean	SD	*F*	*df*	*p*	*η*²
**V**_**UCMlog**_ **vs**. **V**_**ORTlog**_								
	Variance type	V_UCMlog_	−7.42	0.84	1070.03	1;17	<0.001	0.81
		V_ORTlog_	−10.19	1.08				
	Target distance	5 cm	−9.71	1.71	401.95	1.89;32.11	<0.001	0.48
		10 cm	−9.05	1.59				
		20 cm	−8.45	1.47				
		30 cm	−8.01	1.45				
	Movement phase	0–25%	−8.90	1.72	36.67	1.18;20.05	<0.001	0.03
		26–50%	−8.67	1.61				
		51–75%	−8.71	1.63				
		76–100%	−8.96	1.75				
	Variance type * Target distance				20.12	2.28;38.79	<0.001	0.03
	Variance type * Movement phase				93.55	1.47;24.97	<0.001	0.02
	ID * Movement phase				28.45	2.89;49.19	<0.001	0.02
**V**_**UCMlog**_								
	Target distance	5 cm	−8.14	0.70	129.21	1.91;32.41	<0.001	0.39
		10 cm	−7.62	0.69				
		20 cm	−7.17	0.61				
		30 cm	−6.76	0.63				
**V**_**ORTlog**_								
	Target distance	5 cm	−11.27	0.70	392.17	3;51	<0.001	
		10 cm	−10.47	0.72				
		20 cm	−9.73	0.80				
		30 cm	−9.27	0.82				
	Movement phase	0–25%	−10.36	0.98	58.00	1.26;21.49	<0.001	0.10
		26–50%	−9.92	1.14				
		51–75%	−10.00	1.13				
		76–100%	−10.46	0.95				
	ID * Movement phase				27.36	4.19;71.20	<0.001	0.05

The separate analyses of V_UCMlog_ and V_ORTlog_ showed that both variables were larger during pointing movement involving longer target distances (Fig. 2, Table 1, post-hoc pair-wise comparisons all *p* < 0.001 for both variables). Only for V_ORTlog_, an effect of movement phase was found, indicating that this variable was lower in the first and last phase of the movement, as compared to the middle phases (Fig. 2, Table 1, post-hoc pair-wise comparisons all *p* < 0.05). This effect was stronger in pointing movements involving higher IDs, as compared to lower IDs (Fig. 2, Table 1). No other effects were significant. Together, these results showed that joint-angles were linked in synergies so as to stabilise all pointing movements and that the degree of joint-angle variance—both in V_UCMlog_ as V_ORTlog_—was larger for longer target distances. Only V_ORTlog_ showed lower variances at the beginning and the end of the movements, indicating that the end-effector was stabilised more when this was required—i.e. when the end-effector was located near the target.

#### Synergy comparison across task constraints

We chose to base the judgement of whether different synergies were used to stabilise the end-effector movement across different task constraints on whether the joint-angle configurations describing the synergies emerged at a different location in joint-space. To this end, we examined whether joint-angle configurations that participants used across IDs for a certain target distance had a different location in both null space (the subspace in joint-space from which V_UCM_ is computed) and range space (the subspace in joint-space from which V_ORT_ is computed). Using an adapted version of motor equivalence analysis^33,46,47^, for every condition, a synergy boundary demarcating the location of joint-angle configurations was calculated based on projection lengths of joint-angle configurations on both null and range space (see method section for techniques used to determine synergy boundary). Subsequently, the location in both null and range space of joint-angle configurations of other conditions was determined by projecting these joint-angle configurations on both spaces. If the projection length of these joint-angle configurations exceeded the previously defined synergy boundary, joint-angle configurations of the compared conditions emerged at statistically different locations in joint-space—interpreted in the current study as the emergence of different synergies. Note that it was not meaningful to make this comparison across target distances, because end-effector positions, and thus null and range spaces, were different per definition.

**Figure 3:**
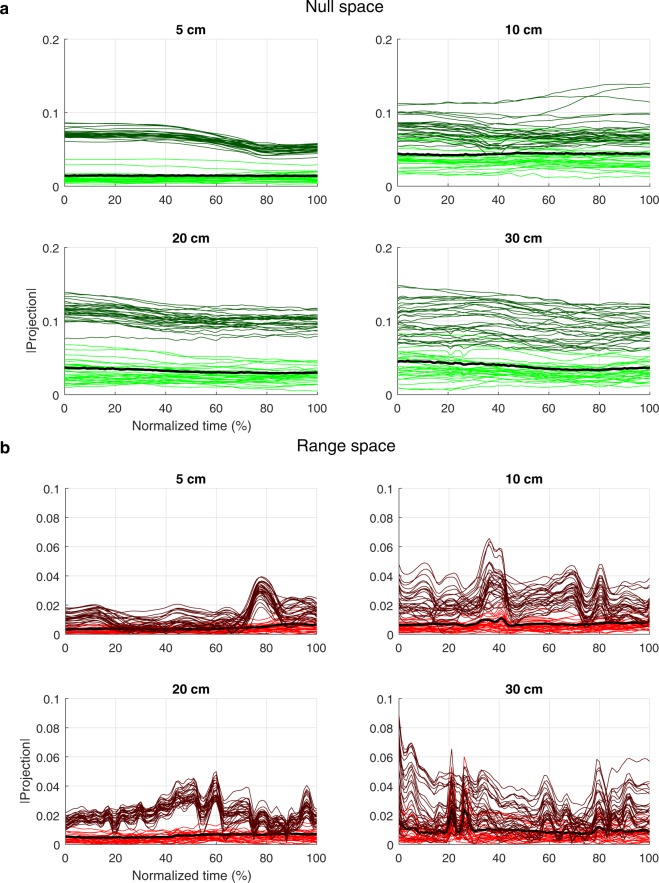
Example of the projection lengths across time for two compared IDs (ID3.5 and ID4) at every target distance in both null space **(a**) and range space. **(b)** Lighter lines indicate the projection lengths across time from the ID (in this example ID3.5) from which the synergy boundary was derived (synergy boundary indicated by the black, bold, line), darker lines indicate the projection lengths of the ID (in this example ID4) from which the projection lengths were compared with this synergy boundary.

Results showed that in both null and range space, the large majority of joint-angle configurations of a certain ID was located outside the synergy boundary of other IDs (Table 2; see Fig. 3 for an example of the graphical representation of differences in projection lengths of joint-angle configurations on both null and range space). This result showed that different joint-angle configurations were used across IDs within one target distance—in the current study interpreted as the emergence of different synergies. In general, joint-angle configurations were more often located outside the synergy boundary in the null space than in the range space (Table 2, indicated by non-overlapping CIs). Furthermore, in both the null and range space, for both cycle halves, joint-angle configurations gradually seemed to be more often located outside the synergy boundary for shorter target distances (Table 2). Medians and ranges across participants, IDs, and time were in the vicinity of the mean percentages as estimated in every space-cycle half-target distance combination (Table 2), indicating that the effects as described above were similar across participants, IDs, and time. Together, these results showed that joint-angle coordination in synergies differed across IDs at every target distance so as to stabilise differences in end-effector movement as observed across these IDs—a result suggesting a reciprocal relation between end-effector and joint-angles.

**Table 2:** Averages [95% CI around the average] (across participants, IDs, and time) for every target distance, and medians [ranges] across participants, IDs, and time of the percentage of joint-angle configurations that were located outside the synergy boundary of other IDs within a target distance.

		First cycle half			
		5 cm	10 cm	20 cm	30 cm
Null space	Mean [CI]	99.3 [99.3–99.4]%	97.9 [97.8–97.9]%	95.5 [95.4–95.6]%	93.6 [93.5–93.7]%
	Median [range] across participants	99.9 [97.0–100]%	98.4 [92.6–100]%	96.4 [87.2–100]%	94.5 [82.4–99.8]%
	Median [range] across IDs	99.4 [98.6–99.9]%	97.9 [97.0–98.8]%	95.2 [93.7–97.5]%	93.3 [90.8–97.2]%
	Median [range] across time	99.3 [99.1–99.6]%	98.0 [97.3–98.2]%	95.4 [94.6–96.4]%	93.6 [92.5–94.6]%
Range space	Mean [CI]	96.4 [96.3–96.5]%	91.7 [91.5–91.8]%	83.4 [83.2–83.6]%	79.1 [78.8–79.2]%
	Median [range] across participants	97.0 [90.7–99.1]%	92.7 [77.8–97.4]%	85.4 [70.9–96.1]%	79.1 [67.3–90.7]%
	Median [range] across IDs	96.5 [95.1–97.5]%	91.9 [89.4–93.0]%	83.3 [82.5–84.3]%	79.4 [76.2–81.7]%
	Median [range] across time	96.5 [94.2–97.7]%	92.7 [88.4–94.0]%	84.3 [76.1–87.7]%	80.8 [69.1–85.8]%
		**Second cycle half**			
		**5 cm**	**10 cm**	**20 cm**	**30 cm**
Null space	Mean [CI]	99.3 [99.2–99.3]%	97.8 [97.7–97.9]%	94.4 [94.2–94.5]%	93.1 [93.0–93.3]%
	Median [range] across participants	99.2 [96.6–100]%	98.4 [92.3–100]%	95.8 [83.0–99.9]%	94.0 [83.6–99.3]%
	Median [range] across IDs	99.3 [98.5–99.9]%	98.0 [96.9–98.6]%	94.4 [92.2–96.3]%	93.1 [90.0–96.7]%
	Median [range] across time	99.3 [99.0–99.5]%	97.8 [97.2–98.4]%	95.0 [91.9–96.1]%	93.5 [91.4–94.1]%
Range space	Mean [CI]	96.4 [96.4–96.6]%	92.3 [92.2–92.5]%	83.5 [83.3–83.7]%	78.5 [78.3–78.7]%
	Median [range] across participants	96.6 [91.6–99.2]%	92.2 [86.0–97.0]%	88.0 [70.3–93.2]%	78.9 [68.5–89.5]%
	Median [range] across IDs	96.8 [95.1–97.4]%	92.5 [90.7–93.1]%	83.6 [83.1–83.8]%	78.5 [75.2–81.2]%
	Median [range] across time	96.5 [94.4–98.2]%	92.7 [89.3–94.3]%	85.0 [77.7–87.6]%	80.0 [70.2–84.4]%

### Do joint-angles have different roles during different end-effector movements across task constraints?

#### Coupling between end-effector and joint-angles

To assess whether joint-angles had a different role in the synergies that stabilised the end-effector movement across task constraints, we took an innovative approach by assessing the coupling between individual joint-angle time-series and the end-effector time-series across conditions using cross-recurrence quantification analysis^48,49^ (CRQA). This analysis quantifies the shared activity of two time-series of their reconstructed state spaces that describe the dynamics of both series. The coupling of end-effector and individual joint-angle movements will present itself in the shared activity of their reconstructed state spaces. To evaluate whether this was the case, we determined for every end-effector-joint-angle combination i), the percentage of recurrences in the reconstructed state spaces (%CREC), ii) the percentage of recurrences that exhibit a deterministic structure (%CDET), and iii) the maximal length of consecutive states in the reconstructed state spaces (CL_max_) as a measure of strength of the coupling between the time-series. This last measure was normalised to the time that the movement cycles within an ID lasted (CL_maxNorm_), to prevent effects on this measure residing from longer movement times, instead of a stronger coupling (as differences in ID severely affected movement time across conditions).

**Figure 4:**
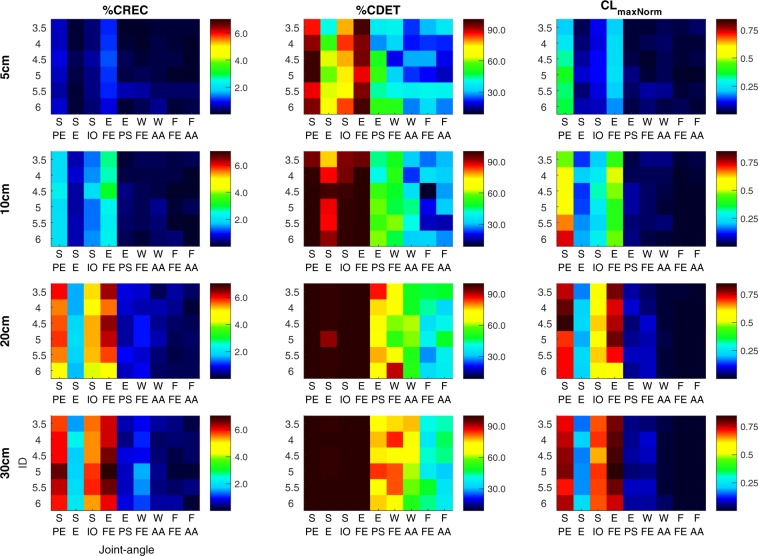
CRQA measures %CREC (left column panels), %CDET (middle column panels), and CL_maxNorm_ (right column panels) across IDs (rows per panel) and distances (row panels) for the CRQA analyses examining the coupling between end-effector and joint-angle time-series. Colours, as presented in the figure, are linked to a certain value of the variable, see colour bar next to panels for colour scaling with respect to variable values. Abbreviated labels S PE: shoulder plane of elevation; S E: shoulder elevation; S IO: shoulder inward-outward rotation; E FE: elbow flexion-extension; E PS: elbow pronation-supination; W FE: wrist flexion-extension; W AA: wrist abduction-adduction; F FE: finger flexion-extension; F AA: finger abduction-adduction.

Results showed similar absolute values of CRQA measures across IDs within each target distance for every joint-angle (Fig. 4), indicating that joint-angle movements differed alongside with the differences in end-effector movements across these IDs. In every condition, the end-effector movement was coupled stronger with three joint-angles (shoulder plane of elevation, shoulder inward-outward rotation, and elbow flexion-extension) than with the other six joint-angles (Fig. 4). These joint-angles had more recurrent states with the end-effector than the other six joint-angles, and the shared states were more deterministic and consisted of longer consecutive trajectories in the reconstructed state space (Fig. 4). Interestingly, the values of the CRQA measures correlated well across IDs within each target distance (Fig. 4, mean [SD] correlation 5 cm: %CREC: 0.61 [0.17], %CDET: 0.91 [0.07], CL_maxNorm_: 0.78 [0.12]; 10 cm: %CREC: 0.87 [0.07], %CDET: 0.96 [0.03], CL_maxNorm_: 0.94 [0.05]; 20 cm: %CREC: 0.95 [0.04], %CDET: 0.98 [0.02], CL_maxNorm_: 0.97 [0.02]; 30 cm: %CREC: 0.98 [0.02], %CDET: 0.96 [0.03], CL_maxNorm_: 0.97 [0.01]). This showed that the relative coupling among joint-angles with the end-effector was similar across IDs. Furthermore, CRQA measures were generally larger in pointing movements involving longer target distances (indicated by the different colour scaling across target distances, Fig. 4); note however that RQA measures of the end-effector (comparing the end-effector time-series with itself) also were larger for longer target distances (mean [ID] %REC: 5 cm: 2.0 [1.2]%, 10 cm: 3.3 [2.0]%, 20 cm: 7.2 [2.7]%, 30 cm: 7.7 [2.8]%; %DET: 5 cm: 98.1 [1.0]%, 10 cm: 99.4 [0.3]%, 20 cm: 99.9 [0.6]%, 30 cm: 99.9 [0.3]%; CL_maxNorm_: 5 cm: 0.21 [0.11], 10 cm: 0.34 [0.17], 20 cm: 0.56 [0.22], 30 cm: 0.52 [0.20]). The increase in CRQA measures thus most likely reflects differences in the stability of the dynamics in the reconstructed state space rather than a difference in coupling strength between the end-effector and joint-angle movement across target distances.

#### Coupling between joint-angles

**Figure 5:**
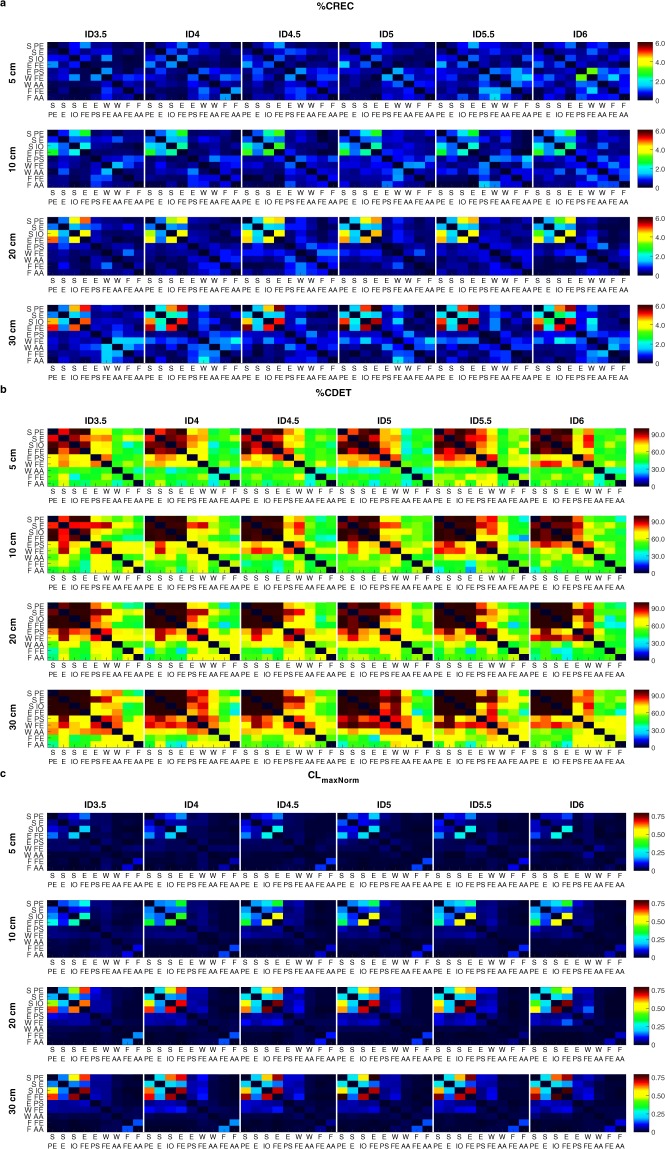
CRQA measures %CREC **(a)**, %CDET **(b)**, and CL_maxNorm_ **(c)** across IDs (columns) and distances (rows) for the CRQA analyses examining the coupling between joint-angle time-series. Colours, as presented in the figure, are linked to a certain value of the variable, see colour bar next to panels for colour scaling with respect to variable values. Abbreviated labels S PE: shoulder plane of elevation; S E: shoulder elevation; S IO: shoulder inward-outward rotation; E FE: elbow flexion-extension; E PS: elbow pronation-supination; W FE: wrist flexion-extension; W AA: wrist abduction-adduction; F FE: finger flexion-extension; F AA: finger abduction-adduction. Diagonals (i.e. the RQA analysis of a joint-angle time-series with itself) were not conducted and set to zero.

To further assess whether the relative coupling among joint-angles in synergies remained similar across IDs, we performed the CRQA analysis again, but now between all possible pairs of joint-angles. The results of these analyses showed that the joint-angles that were coupled stronger with the end-effector movement—i.e. shoulder plane of elevation, shoulder inward-outward rotation, elbow flexion-extension—were also coupled stronger with each other than with the other joint-angles (Fig. 5). Interestingly, no clear groups of joint-angles that coupled with each other could be made among the remaining six joint-angles. Furthermore—in line with the CRQA involving the end-effector movement—values of CRQA measures generally correlated well across IDs within each target distance (Fig. 5, mean [SD] correlation 5 cm: %CREC: 0.65 [0.09], %CDET: 0.89 [0.04], CL_maxNorm_: 0.81 [0.05]; 10 cm: %CREC: 0.64 [0.07], %CDET: 0.85 [0.04], CL_maxNorm_: 0.88 [0.04]; 20 cm: %CREC: 0.66 [0.11], %CDET: 0.94 [0.02], CL_maxNorm_: 0.93 [0.02]; 30 cm: %CREC: 0.81 [0.08], %CDET: 0.88 [0.06], CL_maxNorm_: 0.94 [0.02]), indicating that the relative coupling between joint-angles was similar across IDs. Last, also in these analyses, values of CRQA measures were generally higher for longer target distances (Fig. 5). Together, the results of the CRQA analyses demonstrated that the relative coupling across joint-angles with the end-effector and among each other remained similar across different IDs, indicating that joint-angles had a similar role in the synergies as found across these IDs. Furthermore, results showed that three joint-angles—i.e. shoulder plane of elevation, shoulder inward-outward rotation, elbow flexion-extension—were coupled stronger with the end-effector and with each other than the other six joint-angles, suggesting that these joint-angles had a driving role during the production of the end-effector movement.

#### Contribution of joint-angles to covariation stabilising end-effector

Last, we asked whether the three driving joint-angles had a different contribution—as compared to the remaining six joint-angles—to the covariation among joint-angles in the emerged synergy. Therefore, we evaluated the contribution of the covariation of these three joint-angles to the stabilisation of the end-effector as compared to the remaining six joint-angles. To do this, we assessed the effect of removing the covariation of joint-angles from the synergy on the task variability (TV) at the end-effector, by computing the TV before and after removal of covariation, and subsequently determining the ratio between the two TVs (TV_ratio_; if TV_ratio_ < 1 the removed covariation contributed to the stabilisation of the end-effector)^37,50,51^. We did this in three sets of joint-angles: i) the three driving joint-angles, ii) the six remaining joint-angles, and iii) every combination of three of the remaining joint-angles (to achieve a set with an equal number of joint-angles as the driving joint-angles). From this last option, we took the average across all possible combinations to assess the mean effect. As TV_ratio_ was nonnormally distributed, the logarithmic transform (TV_ratioLog_) was analysed statistically.

**Figure 6:**
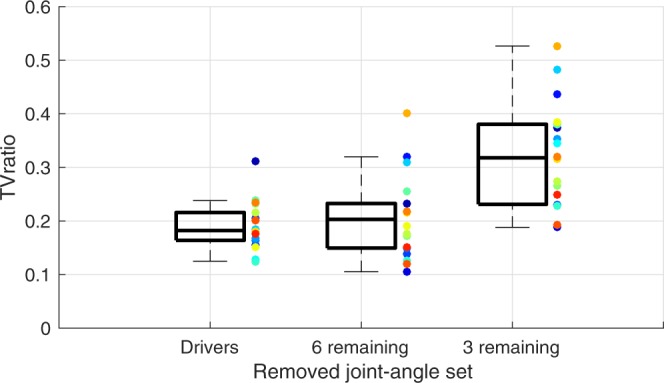
TV_ratio_ per removed joint-angle set, as averaged across cycle halves, IDs, target distances, and movement phases. The boxplot on the right-hand side presents the mean of every possible combination of three of the remaining joint-angles. Coloured dots represent data of individual participants.

For all three joint-angle sets, in every condition, the TV_ratioLog_ was lower than 0 (*p* < 0.001; equivalent to TV_ratio_ lower than 1, Fig. 6), indicating all joint-angles always exhibited covariation with other joint-angles that was important for the stabilisation of the end-effector. Yet, the removal of the covariation of the three driving joint-angles, the six remaining joint-angles, and the mean of every combination of three of the remaining joint-angles yielded for different effects on the TV_ratioLog_ (Fig. 6, F_1.11;18.90_ = 31.04, *p* < 0.001, *η*²_G_ = 0.13; mean [SD]: 3 driving joints = −1.91 [0.14], 6 remaining = −1.96 [0.18], 3 remaining = −1.35 [0.20]). Post-hoc pair-wise comparisons showed that the mean TV_ratioLog_ of every combination of three remaining joint-angles was significantly higher than the TV_ratioLog_ after removal of covariation of either the three driving joint-angles or all six remaining joint-angles (*p* < 0.001). This result indicated that the three driving joint-angles contributed as much as the other six joint-angles to the stabilisation of the end-effector. Furthermore, TV_ratioLog_ was higher for longer target distances (F_3;51_ = 7.49, *p* < 0.001, *η*²_G_ = 0.04; mean [SD]: 5 cm = −1.94 [0.19], 10 cm = −1.81 [0.18], 20 cm = −1.59 [0.20], 30 cm = −1.61 [0.17]), with a significant post-hoc effect between 5 cm, and 20 cm and 30 cm (*p* < 0.005). Furthermore, TV_ratioLog_ was lower for lower IDs (F_5;85_ = 5.94, *p* < 0.001, *η*²_G_ = 0.03; mean [SD]: ID3.5 = −1.85 [0.16], ID4 = −1.85 [0.15], ID4.5 = −1.80 [0.17], ID5 = −1.74 [0.17], ID5.5 = −1.70 [0.18], ID6 = −1.50 [0.25]. Post-hoc pair-wise comparison: ID3.5-4.5 different from ID6, *p* < 0.05) and the first and last movement phase (F_1.25;21.31_ = 100.69, *p* < 0.001, *η*²_G_ = 0.10; mean [SD]: 0-25% = −1.95 [0.14], 26–50% = −1.46 [0.22], 51–75% = −1.55 [0.20], 76–100% = −2.00 [0.13]. Post-hoc pair-wise comparison: all phases different *p* < 0.05). The interaction between ID and movement phase showed that joint-angles contributed less to covariation in the middle phases of the movement for higher IDs, whereas the contribution was equal across IDs for the first and last phase of the movement (F_4.09;69.45_ = 38.88, *p* < 0.001, *η*²_G_ = 0.03). No other effects were significant, indicating that the relative contribution to covariation of a certain joint-angle set was similar across conditions. Together, the results of this indicated that all joint-angles were linked in a synergy that constrained joint-angles so as to covary to stabilise the end-effector movement in every condition.

## Discussion

Synergies that link DOF so as to produce end-effector movements are assumed to reciprocally relate end-effector movement and DOF coordination^cf.^^3,5,9,10^. In the current study, we assessed this reciprocal relation by examining whether, during rhythmic pointing movements, differences in end-effector movement across task constraints—i.e. different IDs based on combinations of target widths and target distances—were related to the coordination of joint-angles in synergies. Results showed that joint-angle configurations that stabilised the end-effector movement in a certain ID were located outside the synergy boundaries of other IDs within every target distance. As these synergy boundaries demarcated the location in joint-space at which joint-angle configurations were found, this finding showed that joint-angle configurations emerged at different locations in joint-space across IDs within a target distance. In the current study, we chose to interpret this finding as the emergence of different synergies based on the differences in task constraints across conditions. Yet, the same three joint-angles—i.e. shoulder plane of elevation, shoulder inward-outward rotation, elbow flexion-extension—had a driving role during the production of the end-effector movement in all conditions, indicating that the differences in joint-angle coordination between synergies were rather small adjustments. Furthermore, all joint-angles—independent of whether they were more drivers of the end-effector movement or were less coupled to the end-effector movement—contributed to the covariation among joint-angles that stabilised the end-effector. Together, results demonstrated synergies that produced the end-effector movement, constrained joint-angles so that they covaried to stabilise the end-effector, and differed when end-effector movement differed. Hence, end-effector and joint-angles were reciprocally related in synergies—indicating that the action system was organised as a complex dynamical system.

The results of the current study are in line with previous studies which found differences in the connectivity among muscles^40,52^ or in low-dimensional structures describing muscle activation patterns^53,54^ across conditions with different task constraints. Together with the finding of different synergies in the current study, these results indicate that the linking of DOF in synergies is temporary, and can be adjusted so as to stabilise the end-effector while meeting the constraints at hand^1,11–13^. In line with this idea, Vernooij *et al*.^39^ showed classical signs of a transition between states—i.e. destabilising synergies before the emergence of a new synergy—across task constraints. At the same time, the fact that synergies emerged at different locations in joint-space while joint-angle roles were similar across task constraints indicated that the differences in synergies were small adjustments as compared to the possible total reorganisation of the links within a synergy—e.g. joint-angles taking new roles. In line with the finding of Vernooij *et al*.^39^, we think that synergies take the form of attractors in a dynamical system that emerge from self-organising processes of interactions between different types of constraints^10,18,19^. Within this paradigm, we suggest that the current results reflect the parameterisation of the attractor based on the specifics of every condition, leading to the emergence of synergies at different locations in joint-space.

The finding that joint-angle roles were rather independent of conditions might indicate that the differences in task constraints across conditions did not invite for joint-angles taking different roles in different synergies across conditions. That is, the task—moving the end-effector between two stationary targets—did not particularly invite to produce the end-effector movements much different than flexing and extending the elbow with accompanying shoulder joint rotations. Alternatively, the emergence of similar roles of joint-angles across conditions might indicate that part of the linkages of DOF have an anatomical nature. Kerkman *et al*.^40^ found that the functional connectivity between muscles is strongly influenced by anatomical constraints—suggesting that the linking of DOF in synergies is supported by the anatomy of the individual^cf.^^4^—while at the same time this connectivity between muscles changes alongside with task constraints. The way DOF are linked in synergies is then the result of a close interplay between the anatomical structure of the action system (acting as constraints in the individual) and constraints in task and environment^11^. Applying this rationale to the current results, constraints in the individual might have played an important part in the basic linking among joint-angles in synergies whereas the different task constraints may have led to the small adjustments of the roles of joint-angles in different synergies. Whether such an interplay between anatomical and task constraints in the linking of DOF in synergies is viable is left to examination at a larger range of task constraints.

Importantly, although joint-angles had similar roles, joint-angle configurations emerged at different locations in joint-space across conditions, showing that differences in end-effector movement were accompanied by differences in the linking of joint-angles in synergies stabilising this end-effector movement. This result indicated a reciprocal relation between end-effector and joint-angles, which was further substantiated in the finding that joint-angles that drove the movement of the end-effector at the same time contributed—together with all other joint-angles—to covariation that stabilised the end-effector movement. Together, these results showed that the end-effector movement emerged from the coordination among joint-angles in a synergy, while at the same time this synergy constrained joint-angles so that they covaried to stabilise the end-effector. This is a key signature of a reciprocal relation between two levels—in this case between end-effector and joint-angles—which is in line with an organisation as expected from a complex dynamical system^cf.^^9,55^. The fact that this reciprocal relation between end-effector and joint-angles was found at every target distance suggests that similar principles of synergy formation are at play at these different target distances and that the influence of task constraints is as important as any other constraint. Differences in synergies as observed across conditions then emerge from the difference in task constraints in interaction with all other constraints. The interaction among constraints then forms, via self-organisation processes, the basis for the coordination of movements^1,11–13^. Since a reciprocal relation and adjustment of synergies based on different constraints can be expected from the coordination of a complex dynamical system, the findings of the current study together suggest that the action system is organised as such a dynamical system during the production of rhythmic pointing movements.

We also found that joint-angles covaried in synergies during the production of rhythmic pointing movements, which is in agreement with studies that assessed the covarying structure in joint-angle variability in discrete pointing movements^27–38^. Thus, interestingly, although previous studies indicated differences in the control of discrete and rhythmic movements^24–26^—e.g. in brain areas involved in either of such movements^25^—the general way joint-angles covary to stabilise the end-effector is similar for these two types of movement. This might suggest that a general principle for motor control exists for the production of both discrete and rhythmic movements, which incorporates both the similarities as well as the differences between such movements. Several perspectives have suggested that such generality in motor control can be achieved by combining a limited set of movement primitives. For instance, the muscle synergies approach^cf.^^56–58^ lies the basis of primitives in the modular organisation of the spinal cord. Importantly, it is assumed that the characteristics of these modules remain fixed over time. The results of the current study showed that the specifics of synergies differed in pointing movements across different IDs. This finding seems to point at a more flexible character of the linking of DOF in synergies, which is in line with several papers demonstrating that muscle synergies differed across task constraints^cf.^^39,53,54,59–63^. An alternative perspective suggests that the large base of movements can generally characterised as the combination of dynamic primitives^64^. These dynamic primitives take then the form of an attractor, emerging from the dynamics of the system. The current results fit with this line of thinking, as all pointing movements across conditions could be characterised as limit-cycle attractors—which then act as a primitive. Furthermore, the linking of DOF in synergies is suggested to be the consequence of the interaction of dynamic primitives^64^. This idea fits the current results since we showed that end-effector movement was related to the coordination of joint-angles. Importantly, we believe that this relation is not unidirectional, but reciprocal—the end-effector movement that emerges from the coordination of joint-angles in turn constrains the way in which DOF are coordinated in this synergy^cf.^^1,3,5,9,10^. Last, it is important to note that these dynamic primitives focus solely on the phenomenology of motor behaviour^cf.^^64^, and not on the principles underlying these phenomena. Note that the enterprise as outlined in the current study—i.e. studying different levels of analyses and their relation—might open routes to understand these dynamic primitives beyond phenomenology, because the ultimate aim is to understand how such primitives emerge from self-organisation.

In conclusion, the current study showed that joint-angles covaried in synergies to produce stable end-effector movements, while the linking of joint-angles in synergies differed across task constraints. The movement of the end-effector was driven by a few joint-angles while all joint-angles covaried to stabilise the end-effector, implying that all joint-angles involved were coordinated in a synergy that produced the end-effector movement. Therefore, the synergy both emerged from the joint-angle coordination and at the same time constrained joint-angles so that they covaried to stabilise the end-effector, while the specifics of the synergy differed alongside with the end-effector movement across task constraints. Together, these results demonstrated that end-effector and joint-angles were reciprocally related in synergies. This type of organisation is typical for a complex dynamical system and, therefore, our findings suggest that the action system was organised as such a dynamical system during the production of rhythmic pointing movements.

## Methods

### Ethics statement

The local ethics committee of the Center for Human Movement Sciences, University Medical Center Groningen, approved the experimental protocol as used in the current study. The experimental protocol was carried out in accordance with the Declaration of Helsinki. Before inclusion, participants received verbal and written information. Before the start of the experiment, participants read and signed informed consent.

### Participants

Twenty right-handed participants were recruited in the current study. Two participants were excluded because of failed data acquisition during the measurements. The remaining eighteen participants (mean [SD] age of 20.8 [1.9] years, eight men) had no neurological or other health issues, and all had normal or corrected-to-normal sight.

### Experimental set-up

**Figure 7:**
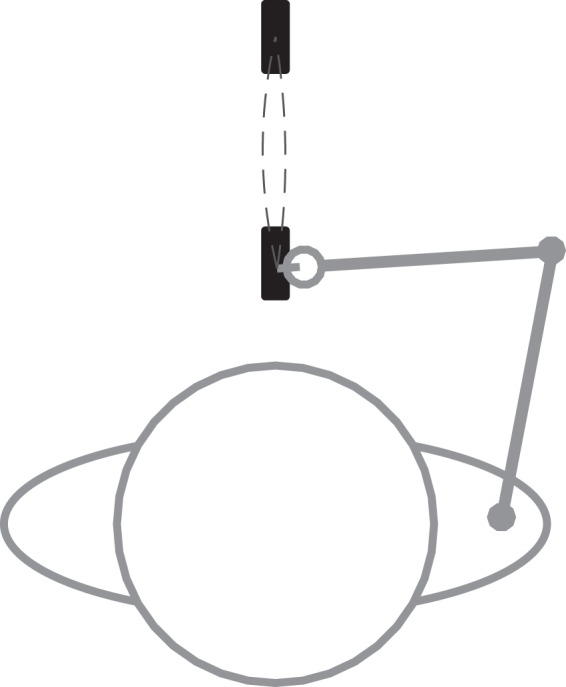
Experimental set-up, as seen from above. Note that the distance between targets (targets indicated by black rectangles) and width of the targets (in the forward-backward direction with respect to the participants heading) was adjusted across conditions. Dashed lines illustrate the end-effector trajectory for one cycle of pointing movements.

Participants made rhythmic pointing movements in a forward direction between two targets following a Fitts’ task paradigm^20,21^ (Fig. 7). We varied ID (3.5–6, with increments of 0.5) and the distance between the targets (5, 10, 20, and 30 cm) across conditions. The various IDs were achieved by varying target width relative to target distance. Targets were presented on a laminated sheet of paper, (A3 size, portrait orientation), which was attached to a table in front of the participants. To avoid drifting of the end-effector in the frontal plane, all targets had a width of 1 cm in this plane (Fig. 7). Participants made the pointing movements with a stylus—leaving no trace at the paper—that was attached to their index finger. The stylus was attached in such a way that it prohibited movement in the interphalangeal joints while allowing for free motion of the metacarpophalangeal joint.

Six rigid bodies, triangular in shape and containing a light-emitting diode (LED) in each of its corners, were attached to the participant’s right side to capture motion data of the pointing movements. One rigid body, which had a leg length of 4 cm, was attached to the stylus; the five other rigid bodies were attached to segments of the participant’s right arm^65^. Two of these rigid bodies, attached to the sternum and the upper arm just below the insertion of the deltoid, had a length of 6 cm; the other three rigid bodies, attached to the dorsal side of the hand, the dorsal side of the upper arm just proximal of the ulnar and radial styloids, and the flat part of the acromion, had a leg length of 4 cm. Movements of the LEDs were captured using two Optotrak 3020 units (Waterloo, Ontario, Canada) which were synchronised and sampled at 100 Hz. To relate the movement of the LEDs to the movement of the participant’s arm, before the start of the experiment, eighteen bony landmarks and the tip of the stylus were digitised using a pointer device^cf.^^65^. Before the start of the experiment, participants were gently strapped against the extended back of a chair using an elastic bandage, to prohibit motion of the trunk during the pointing movements, while allowing for free motion in the shoulder joint.

### Experimental procedure

In every condition, participants made forty cycles of rhythmic pointing movements between the targets presented in front of them. Participants always started the series of cycles with the tip of the stylus in the middle of the target that was presented closest to them (lower target). For this starting point, participants were instructed to take a similar posture across the different IDs for the same target distance. This instruction was repeated before the start of every new set of forty cycles of pointing movements. To check whether participants followed this instruction, we compared the standard deviation of starting joint-angle configurations across IDs within a target distance with the standard deviation of starting joint-angle configurations recorded in previous studies in which a similar starting posture was ensured by means of an elbow placer^29,54^. This comparison showed that participants followed the instruction. During the movements, participants were instructed to move the tip of the stylus as fast and accurate as possible. Experimenters observed whether this instruction was followed, and motivated participants to move as fast as possible while adhering to accuracy demands. Participants were instructed to keep the tip of the stylus at the sheet of paper at all times.

### Experimental design

In total, participants performed rhythmic pointing movements in 24 unique conditions combining every ID with every target distance. Conditions were presented to the participants in a randomised order.

### Data analysis

For each condition, the last thirty consecutive cycles were selected for further analysis. For these thirty cycles, end-effector trajectories were determined from the motion of the three Optotrak LEDs attached to the stylus using rigid body transformations. Also, the trajectories of nine joint-angles—shoulder plane of elevation, shoulder elevation, shoulder inward-outward rotation, elbow flexion-extension, elbow pronation-supination, wrist flexion-extension, wrist abduction-adduction, finger flexion-extension, and finger abduction-adduction—were computed following ISB guidelines for the upper extremity^66^ using segment orientations (derived from the digitised bony landmarks) and the motion of the relevant Optotrak LEDs. These end-effector and joint-angle trajectories were used to derive various measures in the analyses presented in the current study.

#### Does the end-effector movement differ across task constraints?

To construct the phase and Hooke’s portraits for every condition, we computed the average end-effector trajectory in the forward-backward direction in the transversal plane across the thirty selected cycles. End-effector trajectories of individual cycles were first filtered (4^th^ order low-pass Butterworth filter, 5 Hz) and normalised to the median cycle time using a cubic spline before averaging across cycles. From the average trajectory, end-effector velocity and acceleration were derived using numerical differentiation. We used two measures to evaluate the end-effector movement across conditions. These measures were determined for the first and second cycle halves—i.e. the end-effector movements between moments of end-effector movement reversal at the targets—separately in every condition. First, the non-linear component of the end-effector movement was determined by calculating the residual variance of linear regression lines through the trajectory in the Hooke’s portrait: NL = 1 − R^2^_linear_^cf.^^14^. Second, we computed the ratio between acceleration and deceleration times (AD_ratio_) for each individual cycle and subsequently averaged across cycles before entering statistical analysis.

#### Are joint-angles linked in different synergies so as to stabilise differences in end-effector movement across task constraints?

Synergistic linking among joint-angles: To perform the UCM method^43–45^ in the current study, the nine joint-angles were selected as elemental variables, and the 2D position (position in the transversal plane) of the end-effector was selected as the performance variable. To relate changes in joint-angles to changes in the end-effector, for every condition, we constructed a Jacobian matrix (*J*) using linear regression procedures^67^. The null space of *J* reflects a manifold in joint-space that contains all joint-angle configurations that stabilise the end-effector on its mean position, whereas the orthogonal complement of *J*—i.e. the range space of *J*—includes all joint-angle configurations that lead end-effector positions to deviate from its mean position. The UCM method divides the joint-angle variance across cycles in a part that varies along the manifold (V_UCM_) and a part that varies along its orthogonal complement (V_ORT_).

Before entering the UCM method, time-series of the end-effector and joint-angle movements of every individual cycle half were normalised in time (0–100%) using a cubic spline. Subsequently, per condition and for both the first and second cycle half, V_UCM_ and V_ORT_ were computed per instant of normalised time. To do this, we first computed a covariance matrix *C* using the joint-angle configurations across cycles at each instant^50,51^. Subsequently, V_UCM_ and V_ORT_ were computed as:

1 $${V}_{UCM}=\frac{trace(null{(J)}^{T}\,\ast \,C\,\ast \,null(J))}{DOF-DV}$$

2 $${V}_{ORT}=\frac{trace(orth{({J}^{T})}^{T}\,\ast \,C\,\ast \,orth({J}^{T}))}{DV}$$

in which DOF are the nine joint-angles and DV (dimension performance variable) is the 2D position of the end-effector. Last, V_UCM_ and V_ORT_ were averaged across movement phases of 25% (i.e. 0–25%, 26–50%, 51–75%, 76–100%) before entering statistical analysis.

Synergy comparison across task constraints: We used an adapted version of motor equivalence analysis^33,46,47^ to compare the location of joint-angle configurations describing the used synergies in both null and range spaces across task constraints. This was done by determining, for every condition, a synergy boundary demarcating the location of joint-angle configurations in both null and range space. For every participant, every condition, and every instant of normalised time, for both null and range space, we determined this synergy boundary by calculating the projection length of a joint deviation vector (computed as the difference between the joint-angle configuration of a certain cycle and the mean joint-angle configuration across cycles) on both the null and range space. Projection lengths were normalised to the dimension of each of the spaces^cf.^^33^. Subsequently, the synergy boundary was determined as the upper limit of the 95% confidence interval around the mean projection length, which was calculated using bootstrap statistics with resampling of 1000 times.

After the determination of the synergy boundary for every condition, we examined, within every target distance, whether joint-angle configurations as used across cycles involving a certain ID exceeded the synergy boundaries as determined for other IDs. We first determined, for every possible ID comparison and for every instant of normalised time, cross-ID joint deviation vectors (computed as the difference between joint-angle configuration vectors of individual cycles of one ID and the mean joint-angle configuration vector of another ID whose synergy boundary was used), which were subsequently projected onto the null and range spaces of the ID whose synergy boundary was used. Next, projection lengths were again normalised to the dimension of each of the spaces and subsequently compared to the synergy boundary of that ID in each of the subspaces. If the length of this projection systematically exceeded the value of the synergy boundary, we concluded that joint-angle configurations were located at different places in null and/or range space in the two compared IDs—interpreted in the current study as the emergence of different synergies. To determine whether this was generally the case, we determined the percentage of cases with projections that fell outside the synergy boundary of the various conditions. Per subspace, per cycle half, and per target distance, the 95% CI (calculated with bootstrap statistics, 1000 resamples) around the mean across participants, IDs, and normalised movement time was determined to assess whether percentages differed for subspaces, cycle halves, and target distances, whereas we used medians and ranges to determine whether this was generic across participants, IDs and normalised movement time.

#### Do joint-angles have different roles during different end-effector movements across task constraints?

Coupling between end-effector and joint-angles: To perform the CRQA analyses^48,49^ between full end-effector and joint-angle time-series (including all thirty cycles), time-series were first converted to z-scores^cf.^^68,69^ to achieve a common scale for the compared time-series. Subsequently, state spaces were reconstructed for the two compared time-series (i.e. end-effector vs. individual joint-angles). For this state space reconstruction, we first determined time delay and embedding dimension as required input parameters. Time delay was determined as the first minimum in the average mutual information function^70^ applied on both time-series separately. With this time delay, the embedded dimension of both time-series was determined using false nearest neighbours analysis^70,71^. If the found embedding dimension was below 10, we increased the embedded dimension of that time-series to follow recommendations of using embedding dimensions of 10–20 when applying CRQA analysis in biological systems^cf.^^49^. The highest number of dimensions of the compared time-series was selected as the embedded dimension for that comparison. Last, before reconstructing the state space of both time-series, a new time delay, now by applying the average mutual information function on the combination of the two compared time-series, was determined.

With the two reconstructed state spaces, we determined the difference matrix by computing the Euclidian distance between all points of the two state spaces. This difference matrix was converted to a recurrent matrix by applying a radius—which defines what the distance between points in the compared state spaces maximally can be to consider points as recurrent—to the distance matrix. This radius was selected as a percentage of the mean distance between points as defined in the difference matrix. Because CRQA measures were utilised to evaluate the coupling between end-effector and joint-angles in different synergies across IDs within a certain target distance, radii were determined per target distance separately. Following the recommendation that the percentage of recurrences should be kept low (0.1–2%^cf.^^49^), we selected the first radius for which in each comparison between two time-series the lower bound of the 95% CI around the sample mean of the percentage of recurrences—determined across participants and IDs within a certain target distance with bootstrap statistics, 1000 times resampling—exceeded 0.1%. This resulted in the following radii: 5 cm: 21%, 10 cm: 22%, 20 cm: 25%, and 30 cm: 25%. From the recurrent matrix, we determined the CRQA measures percentage of recurrences of the reconstructed state spaces (%CREC), percentage of recurrences that exhibit a deterministic structure (%CDET), and maximal length of consecutive states in the reconstructed state spaces (CL_max_) as a measure of strength of the coupling between the two time-series. This last measure was normalised to the time that the movement cycles within an ID lasted (CL_maxNorm_), to prevent effects on this measure residing from longer movement times, instead of a stronger coupling, in pointing movements involving higher IDs.

Coupling between joint-angles: The same rationale and outcome variables as described above were used to evaluate the coupling among joint-angles, but now with the state space comparison between every possible combination of two joint-angle time-series. Radii for these analyses were selected at: 5 cm: 26%, 10 cm, 26%; 20 cm: 24%, and 30 cm: 25%.

Contribution of joint-angles to covariation stabilising end-effector: Last, we determined the effect of the removal of covariation of joint-angles on task variability (TV), i.e. the 2D end-effector variability^37,50,51^. This TV was calculated as:

3 $$TV=J\ast C\ast {J}^{T}$$

Subsequently, the variability in the 2D end-effector when the covariance of a set of joint-angles (i.e. three driving joint-angles, six remaining joint-angles, and every possible combination of three of the remaining joint-angles) was removed from the covariance matrix *C*, TV_0_ was computed as:

4 $$T{V}_{0}=J\ast {C}_{0}\ast {J}^{T}$$

Subsequently, TV and TV_0_ were united in a ratio (TV_ratio_).

### Statistical analysis

Data of all included participants of all conditions were taken into account in every statistical analysis. Every dependent variable examined in the current study was assessed on its normality with Shapiro-Wilk tests. Only AD_ratio_ exhibited normally distributed data. We used logarithmic transformation^cf.^^72^ to correct for the non-normal distribution in the remaining variables. This transformation was appropriate for the variables NL, V_UCM_, V_ORT_, and TV_ratio_. For these logarithmically transformed variables and AD_ratio_, differences between conditions were assessed with repeated-measures ANOVAs, with target distance, ID, and cycle half, and movement phase (added factor V_UCM_, V_ORT_, and TV_ratio_ analyses), variability type (added factor for joint V_UCM_ vs. V_ORT_ analysis), or removed joint-angle set (added factor for TV_ratio_ analysis) as within-subject variables. If within these ANOVAs the assumption of sphericity was violated, the Greenhouse-Geisser correction was used. All effects were tested with an alpha level of 5%. Effect sizes were established with the generalised eta-squared (*η*^2^_*G*_)^73,74^ using 0.02 as a small effect, 0.13 as a medium effect, and 0.26 as a large effect^74,75^ p.413–414. Only effects larger than 0.02 were taken into consideration. Significant main effects were followed with post-hoc Bonferroni-corrected pair-wise t-tests. Logarithmically transformed values of TV_ratio_ were assessed for their difference from 0 with Bonferroni-corrected one-sample one-tailed t-tests.

Normality of the variables %REC, %DET, and CL_maxNorm_ in both CRQA analyses could not be achieved using logarithmic transforms. Therefore, the similarity in the distribution of CRQA measures across IDs was assessed with Spearman’s rho correlation coefficients for every target distance. All statistical analyses were performed using SPSS version 22 and Matlab version R2016a.

## Data Availability

The datasets generated during and/or analysed during the current study are available in the Figshare repository, doi.org/10.6084/m9.figshare.8280893.
